# Applications of Metals for Bone Regeneration

**DOI:** 10.3390/ijms19030826

**Published:** 2018-03-12

**Authors:** Kristina Glenske, Phil Donkiewicz, Alexander Köwitsch, Nada Milosevic-Oljaca, Patrick Rider, Sven Rofall, Jörg Franke, Ole Jung, Ralf Smeets, Reinhard Schnettler, Sabine Wenisch, Mike Barbeck

**Affiliations:** 1 Clinic of Small Animals, c/o Institute of Veterinary Anatomy, Histology and Embryology, Justus Liebig University of Giessen, D-35392 Giessen, Germany; Kristina.Glenske@vetmed.uni-giessen.de (K.G.); Nada.Milosevic-Oljaca@vetmed.uni-giessen.de (N.M.-O.); 2 Botiss Biomaterials, D-12109 Berlin, Germany; Phil.Donkiewicz@botiss.com (P.D.); alexander.koewitsch@botiss.com (A.K.); patrick.rider@botiss.com (P.R.); Sven.Rofall@botiss.com (S.R.); 3 Clinic for Trauma Surgery and Orthopedics, Elbe Kliniken Stade-Buxtehude, D-21682 Stade, Germany; Joerg.Franke@elbekliniken.de; 4 Department of Oral and Maxillofacial Surgery, University Hospital Hamburg- Eppendorf, D-20246 Hamburg, Germany; ol.jung@uke.de (O.J.); r.smeets@uke.de (R.S.); 5 Justus Liebig University of Giessen, 35390 Giessen, Germany; reiner.schnettler@mac.com

**Keywords:** metals, dental regeneration, bioactivity, tissue regeneration, bone

## Abstract

The regeneration of bone tissue is the main purpose of most therapies in dental medicine. For bone regeneration, calcium phosphate (CaP)-based substitute materials based on natural (allo- and xenografts) and synthetic origins (alloplastic materials) are applied for guiding the regeneration processes. The optimal bone substitute has to act as a substrate for bone ingrowth into a defect, as well as resorb in the time frame needed for complete regeneration up to the condition of *restitution ad integrum*. In this context, the modes of action of CaP-based substitute materials have been frequently investigated, where it has been shown that such materials strongly influence regenerative processes such as osteoblast growth or differentiation and also osteoclastic resorption due to different physicochemical properties of the materials. However, the material characteristics needed for the required ratio between new bone tissue formation and material degradation has not been found, until now. The addition of different substances such as collagen or growth factors and also of different cell types has already been tested but did not allow for sufficient or prompt application. Moreover, metals or metal ions are used differently as a basis or as supplement for different materials in the field of bone regeneration. Moreover, it has already been shown that different metal ions are integral components of bone tissue, playing functional roles in the physiological cellular environment as well as in the course of bone healing. The present review focuses on frequently used metals as integral parts of materials designed for bone regeneration, with the aim to provide an overview of currently existing knowledge about the effects of metals in the field of bone regeneration.

## 1. Introduction

The regeneration of bone is of special interest, most notably in dental medicine. For the regeneration of bone tissue of the jaw and also within the sinus cavity, autografts are still the so-called “gold standard” due to their osteoinductive, osteogenic and osteoconductive regenerative capacities [1]. These properties are based on the different components of the transplanted bone tissue: calcified bone matrix, different bone cell types, i.e., osteoblasts, osteocytes and osteoclasts, and the connective tissue including the vasculature and, thus, endothelial cells, as well as other, different cell types such as macrophages (so-called “osteomacs”) and fibroblasts, amongst others, are components of autografts [2]. Additionally, bone-associated proteins such as members of the bone morphogenetic protein (BMP) family or osteopontin, osteonectin and osteocalcin beside matrix- and cell-related metal ions are integral parts of autografts. An autograft represents a physiologically active transplant, as all of these components allow support of the bone regeneration process after implantation into a defect side [3,4]. However, the application of autografts requires the harvest of healthy bone tissue from another part of the body, i.e., from extraoral locations such as the hip crest or intraoral localizations such as the mandibular ramus. Thus, one of the disadvantages of the application of bony autografts is the second defect side that is created for harvesting of the bone tissue. Besides different complications that could accompany this second surgical intervention, the amount of bone tissue from other locations is often limited and, thus, is not sufficient to fill a bone defect [5].

Beside autografts, a variety of so-called bone substitute materials has been developed within the last decades to overcome the issues with bone autografts. In this context, two main material classes are differentiated: bone substitutes based on “natural” precursors and synthetic materials [6]. The natural-based bone substitute materials are mainly processed from human or animal bone (allo- and xenografts). For the manufacturing of allogenic bone substitutes, bone tissue from living donors, i.e., from femoral heads, or of dead donors, is used, while xenografts are mainly processed from bovine bone (or recently porcine bone). Furthermore, different natural materials based on a variety of biopolymers such as silk fibroin, amongst many others, have been analyzed for application as bone substitutes within the last decades [7,8].

Moreover, different synthetic bone substitute materials have been developed, and most of these materials that are clinically applied are based on calcium phosphates such as hydroxyapatite (HAp) or β tricalcium-phosphate (β-TCP) [9]. Even mixtures of these compounds have been shown to provide good healing results based on the combined degradation behavior. Moreover, a variety of other synthetic materials also combined with techniques such as three-dimensional printing procedures have been tested and have shown to be suitable for bone regeneration [10,11,12]. 

However, the regenerative properties of all the afore-mentioned biomaterials are restricted, particular in comparison to autografts as most of the bone substitute materials provide only a basis for osteoconductive bone growth [13]. Until now, no bone substitute material has been developed that features comparable regenerative capacities compared to autografts. 

Different strategies have been originated to overcome this issue. A first group of concepts includes synthetic bone substitute materials with controllable material characteristics such as porosity or (nano-) topography [14]. It has been suggested that even these special material properties, which are often stated to mimic the characteristics of the bony extracellular and calcified matrix and, thus, being “biomimetic”, allow for induction of bone growth [15]. Interestingly, many publications including in vitro studies and in vivo analyses within ectopic tissues such as the subcutaneous connective tissue, describe osteoinductive properties of especially developed synthetic bone substitute materials [16]. However, the suspected osteoinductive properties of such materials have never been revealed in clinical studies, indicating that such a concept is still not tenable.

A second concept group includes the addition of different biologically active agents such as collagen, hyaluronic acid or osteoinductive molecules, such as members of the bone morphogenetic protein (BMP) family [17,18,19,20]. In this context, it has been shown that the combination of synthetic bone substitutes with extracellular matrix proteins, such as collagen, leads to diverse regenerative results. On the one hand, the polymer addition can allow an increase in bony integration behavior, while other results report significantly lower bone growth rates for such a material composition in comparison to the bone substitute material alone [21,22,23,24]. In the case of the addition of molecules such as BMPs, other issues have been realized, although a variety of studies has shown their exceptional regenerative properties [25,26,27]. This results from the facts that the underlying regenerative mechanisms of BMPs are not yet understood and possible side effects are not well-known, especially since such molecules are usually administered in non-physiological doses (thousands to millions times the amount normally found in the body) [28]. Additionally, such molecules are still very expensive, although also available as recombinant proteins compared to other bone substitute materials [27]. Furthermore, the effect of the immobilized growth factor also depends on the amount released within a certain timeframe. Hence, the material properties such as porosity play a significant role [29]. 

A further group of tissue engineering concepts includes the addition of different cell types to bone substitute materials. Most often osteoblasts and their precursor cells are used for such material-cell-combinations, based on the fact that this cell type is mainly involved in bone regeneration by deposition of the organic extracellular matrix and its subsequent mineralization [30]. In this context, mesenchymal stem cells are also of special interest as this cell type represents the earliest cellular step in osteoblastic differentiation [31]. Furthermore, the additions of other cell types that directly or indirectly support the bone growth process have been examined [32]. For example, the influence of different endothelial cell types, such as human dermal microvascular endothelial cells (HDMEC) in mono- or co-culture with bone substitute materials, have been analyzed to provide fast and sufficient vascularization, which is an important factor for bone tissue regeneration [33,34]. Additionally, blood cells or “inflammatory” cells such as cell types of the monocyte/macrophage line have been used to increase the regenerative properties of bone substitutes [35]. This concept is based on the assumption that such cell types express different molecules that are involved in (bone) tissue healing and might induce or at least increase the process of bone regeneration [36,37,38]. In this context, a broad spectrum of scaffolds combined different blood cells—for example, platelet-rich plasma (PRP) or platelet-rich fibrin (PRF)—obtained by simple centrifugation from freshly drawn venous blood, have also been suggested to increase or even induce bone regeneration [39,40,41]. The assumption of such concepts is that both the obtained cells and moreover growth factors present within the blood, should stimulate (bone) tissue regeneration [42]. However, all of these tissue engineering concepts did not find their way into the clinic as they are either not applicable in acute surgical situations due to the long time spans needed for cell isolation and co-cultivation with a bone substitute, or their clinical efficacy has still not been proven by scientific analyses such as in the case of PRP or PRF concepts.

A further concept is the application or the combination of different metals or metal ions with bone substitute materials in the field of bone regeneration. Different metal ions are essential components of different tissues, such as calcium phosphates for extracellular calcified bone matrix or integral component of cells or proteins that regulate essential cellular processes, including proliferation and differentiation [43,44,45]. Together, the different metal ions have functional roles in the physiological cellular environment as well as in the course of bone healing. Thus, the application of metal ions in combination with the above-mentioned bone substitutes or singularly, is of special interest for bone regeneration [46,47,48]. To provide an overview of the regenerative potential of the different metal ions, the present review summarizes the knowledge about their involvement in cellular processes and the bone healing process, with a further focus on studies that have already analyzed the regenerative potential of bone substitutes, including metals.

## 2. Bone Tissue Healing and Approaches for Material-Related Support

The process of bone tissue healing is based on different factors. Primarily, the bone related cells, i.e., osteoblasts, osteoclasts and their precursors, are involved in this process [49]. In this context, most bone substitute materials allow for the osteoconductive ingrowth of osteoblasts and mesenchymal progenitors acting as a scaffold structure [50,51]. Afterwards, osteoblasts produce the extracellular organic bone matrix, which mainly consists of collagen type 1, and hydroxyapatite is crystallized on the collagen fibrils. Moreover, osteoblasts trigger and promote the crystallization by secretion and expression of various other proteins or receptors such as the receptor activator of NF-κB ligand (RANKL) and GDF5 [52]. Thus, osteoblasts and their precursors are always a first starting point for different concepts that should improve bone healing [53]. Interestingly, different ions such as Mg^2+^ ions influence osteoblastic growth, proliferation or differentiation [54,55]. 

Moreover, influences on bone-resorbing cells and their precursors, i.e., multinucleated osteoclasts and hematopoietic stem cells as well as the different intermediate stages, are of great interest in the field of bone tissue regeneration [56]. This is based on the fact that a molecular cross-talk between osteoblast and osteoclasts has been revealed and additionally, it has been shown that both cell types are organized in so-called bone remodeling units (BRU) or basic multicellular units (BMU) [57,58]. On the one hand, osteoblasts play an important role in osteoclastogenesis and bone resorption based on the expression of different molecules such as the receptor activator of NF-κB ligand (RANKL), the macrophage-colony stimulating factor (M-CSF), interleukin (IL)-1β, IL-6 and IL-11, amongst others [52,59,60]. Furthermore, osteoblasts also express different inhibiting molecules such as osteoprotegerin (OPG), the granulocyte macrophage-colony-stimulating factor (GM-CSF), IL-3, IL-12 and IL-18, which led to the conclusion that a balanced control of bone remodeling is achieved. On the other hand, different coupling factors are nowadays known that are expressed by osteoclasts such as tartrate-resistant acid phosphatase (TRAP), sphingosine 1-phosphate (S1P), bone morphogenetic protein 6 (BMP-6), hepatocyte growth factor (HGF) and collagen triple helix repeat containing 1 (CTHRC1), amongst others, inducing osteoblastic growth or bone formation [61]. Thus, this cell type constitutes a further approach for enhancement of bone regeneration. In this context, it has already been shown that ions such as Sr^2+^ ions can influence bone formation via depression of osteoclast-mediated bone resorption (for further details see paragraph 3).

Additionally, other cell types such as endothelial cells are involved in the process of bone tissue healing, as sufficient vasculature and the related transport of both nutrients and metabolic end products are basic factors for bone formation [33]. Thus, this cell type and functional blood vessels are also a key factor in the regeneration process. In this context, both the process of bone healing and angiogenesis are directly coupled via different local factors [62]. Primarily, the so-called hypoxia-inducible factor 1-α (HIF-1α) pathway is induced by local hypoxia affected by a bone injury as a key mechanism for coupling bone growth to angiogenesis [63]. The induction of this pathway results in an increased expression of the vascular endothelial growth factor (VEGF), one of the most important and strongest angiogenic cytokines, which is also expressed by osteoblasts and cell types such as macrophages [64]. The expression of VEGF leads to blood vessel ingrowth within the defect area and has a direct influence on osteoblast growth and proliferation as well as matrix deposition [64].

Moreover, the connection between the immune system and bone tissue metabolism and regeneration has been recognized in more detail in recent years. In this context, it has been revealed that a special subtype of the macrophage line within bone tissue, so-called osteomacs, is a further key element for bone formation [65]. Interestingly, these osteal macrophages are also integrated into resting bone tissue and are enriched at sites of bone formation combined with the inflammatory process following bone injury [37]. Following their activation, osteomacs have shown to promote osteoblastogenesis and matrix deposition via the nuclear factor (NF)-κB signaling pathway, which is important for their pro-osteogenic function [66]. Furthermore, it has been revealed that a direct cell-cell-contact between osteomacs and osteoblasts takes place, ensuring an osteoblastic maintenance and homeostasis via the sequestosome 1/p62-dependent low-level activity of NF-κB [65,67].

Finally, the reaction of different inflammatory cells to an implanted bone substitute influences the process of bone healing [68,69]. Thus, research in the field of biomaterial-induced inflammation has increasingly focused on bone regeneration research. In this context, it has been revealed in the last decades that nearly all bone substitute materials induce an inflammatory cascade, the so-called “foreign body response to biomaterials”, after its application [70]. In this cascade, the initial accumulation of proteins, which is highly specific for every biomaterial dependent on its respective physicochemical characteristics, causes the further binding of a first generation of different cells and following inductions of specific signaling pathways [71,72]. This first generation of cells within an implantation bed guide the cellular processes via expression of different molecules or cytokines [73]. Interestingly, it has been revealed that in this inflammatory cascade, macrophages and their fused end stages, the so-called multinucleated giant cells (MNGCs), are key cellular components [74,75]. Those cell types have shown to express both pro- and anti-inflammatory molecules such as VEGF that guide the integration behavior and factors such as the implant bed vascularization of bone substitute materials [64,76]. Additionally, other cell types such as granulocytes or thrombocytes have been partially revealed to have an eminent influence on this tissue reaction cascade, which finally leads to different outcomes of the bone regeneration process. Material factors, for example, the chemical composition or physical material properties such as the porosity or the surface structure, as well as the involvement of different ions such as Cu^2+^ ions, have been shown to influence the inflammatory tissue reaction caused by a bone substitute material [71,77,78].

## 3. Metal Ions, Their Physiological Functionalities and Role in Bone Healing

**Figure 1:**
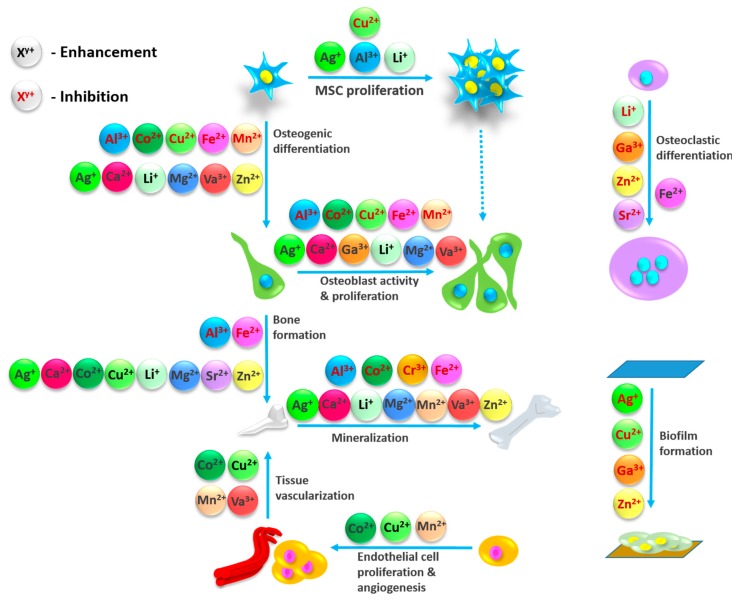
Influence of metal ions on the variety of processes involved in bone regeneration.

Metals have been widely accepted as implant materials for a few decades. Even when a solid metal is applied to a physiological environment, it is always in an equilibrium with its ions. These metal ions are responsible for a variety of biochemical functions, which are important for the different steps of bone regeneration, as they influence the equilibrium between osteoblasts, osteoclasts and osteocytes. Thus, metals and their corresponding ions, which have an influence on the process of bone healing, should be mentioned here (Figure 1). We will also outline the impact on different states of tissue formation and the interplay between related metal ions in processes leading to bone regeneration.

### 3.1. Aluminum (Al^3+^)

Aluminum does not belong to the group of trace elements, is not involved in any physiological functions and is consequently not essential for the human organism [79]. Intake of elevated quantities of aluminum is associated with toxic effects leading to serious adverse reactions including anemia, encephalopathy and osteoporosis, as Al^3+^ ions compete with essential ions like Fe^2+^ [80,81,82]. Investigation of the specific reaction of human neural cells to aluminum exposure, for example, showed that concentrations as little as 100 nM of aluminum sulfate significantly elevated atypical, pro-inflammatory and pro-apoptotic gene expression [83].

Reports about the functions of aluminum in bone formation are ambiguous. Positive impacts of aluminum supplementation on osteogenesis in beagles as well as in osteopenic rats was previously demonstrated, initiating a further interest in the investigation of aluminum in tissue engineering [84,85]. In contrast to these findings, expression of osteoblast activity markers was substantially lower, while expression of apoptotic markers was increased when treated with aluminum, demonstrating impaired cellular activity and survival, and a clear link between aluminum intoxication and compromised bone formation [86]. Quarles and colleagues put their findings on the positive impact of aluminum on de novo bone formation into context with the contradicting literature and suggested that aside from discrepancies in the applied model/organism, aluminum concentrations and time of exposure, a paradoxical impact of aluminum on mesenchymal progenitors and mature osteoblasts could be the main reason for these dissimilar observations [85]. This hypothesis was further supported by another group, which demonstrated that aluminum ions provoked a chemotrophic stimulus in preosteoblasts while having an inhibitory effect on osteoblasts [87]. 

Negative impacts of aluminum on osteoblast function, however, are prevalent in the contemporary literature. An in vivo study in rats assessed the effects of aluminum exposure on the uptake of bone mineral elements, trace elements and bone mineral density. The levels of analyzed trace elements were significantly lower with aluminum exposure, and deposition of calcium, phosphorus and magnesium was decreased in comparison to the control population. Bone mineral density in the femur metaphysis of the aluminum-treated group was also significantly lower compared to the control, resulting in pronounced bone loss [88]. Aluminum does not seem to contribute to bone and tissue healing but to have a rather opposing impact on this process so that, aside from favorable mechanical properties, addition of aluminum to implantable medical devices offers no scientifically evident benefits. Furthermore, other bioceramics such as zirconia oxide are progressively emerging as bioinert alternative to aluminum oxide [89].

### 3.2. Calcium (Ca^2+^)

Calcium is an important functional component of biodegradable calcium phosphate-based biomaterials designated for bone regeneration in orthopedics, trauma surgery, and in dentistry (for reviews: [90,91,92]). 

Calcium is the most common mineral of the body and is primarily stored in the skeleton [93]. Calcium homeostasis is tightly regulated by the parathyroid hormone (PTH) and calcitonin, which regulate calcium serum levels by stimulating (PTH) or inhibiting (calcitonin) bone resorption mediated by osteoclasts. During bone remodeling, bone resorbing osteoclasts can create local concentrations of extracellular calcium ions up to 40 mM [94]. These microenvironmental increases are known to inhibit resorption activity of osteoclasts and to stimulate proliferation and differentiation of mesenchymal stromal cells [93,95,96,97,98,99] and osteoblasts [100,101]. 

During the 1980s, extracellular calcium was shown to activate an extracellular G-protein-coupled receptor, termed the calcium sensor receptor (CaSR) [102]. The CaSR is expressed in cells of the hematopoietic lineage, such as in monocytes [103], osteoclasts [104] and in cells of the mesenchymal lineage [93,99,101,105,106]. Regarding the high responsiveness of bone cells to extracellular calcium, elevated levels of calcium enhance proliferation chemotaxis and osteogenic differentiation of bone marrow-derived mesenchymal stromal cells in a dose-dependent manner by activating the CaSR [93,99]. Downstream, the intracellular pathway induces phosphorylation of extracellular signal-regulated protein kinases 1 and 2 (ERK 1/2) [99], which are part of the mitogen-activated protein kinase (MAPK) signaling pathway, playing an important role in regulating cell proliferation in various mammalian cells [107]. The activation of the CaSR in response to extracellular calcium levels also stimulates phospholipase C (PLC) and induces sustained increase of cytosolic calcium in rat calvarial osteoblasts [106]. The activation of PLC results in generation of inositol 1,4,5-trisphosphate (IP3) and triggers IP3-receptor-mediated calcium release from the endoplasmic reticulum. As a result, store operated calcium entry (SOCE) mediates extracellular calcium entry into the cells for endoplasmic reticulum-calcium store filling [108]. In addition to the effects mediated by the CaSR and the SOCE route, voltage gated calcium channels may also serve as structural units accounting for calcium entry into osteoblasts [106], and osteogenic differentiation of osteoprogenitors [96,109].

Given the superior significance to modulate cellular functions, variations in extracellular calcium in the milimolar range result in proliferation, survival and chemotaxis as well as in differentiation of osteoblasts [101,106] and bone marrow-derived mesenchymal stromal cells (MSCs) [93,96,99]. Optimal conditions to stimulate proliferation of rat calvarial osteoblasts include extracellular calcium concentrations in the range of 3 and 10 mM [101]. Proliferation of bone marrow-derived MSCs harvested from different species (i.e., human, porcine, rat) is effectively supported by concentrations of 4 mM [99], 7.8 mM [96], and 10 mM [93]. Additionally, osteogenic differentiation capacity of human bone-derived MSCs is stimulated in response to extracellular calcium concentrations in the range of 10 and 20 mM [98].

According to the composition of natural bone and the pivotal role of calcium in cellular functions, various calcium phosphate-based materials have been developed for bone replacement therapies [90,91,92]. Incorporation of the calcium phosphate phases modulates bioactivity of the biomaterials, and as pointed out in previous studies, high bioactivity is equivalent to calcium phosphate binding capacity and causes depletion of calcium in close vicinity to the biomaterial [97,104,110]. Calcium phosphate deposition along the surface of bone substitute materials represents an advantageous property to support osseointegration. However, the calcium-deficient microenvironment in close vicinity to the materials remains obscure—especially considering the aforementioned calcium-dependent effects on osteoblasts and progenitor cells. It has been shown that osteoprogenitors—as in the case of bone-derived MSCs—can overcome calcium deficiency when they are cultured in combination with highly bioactive xerogels [97]. The mechanism by which the cells maintain their functional integrity even in response to calcium levels close to zero is still not clear. Given the fact that the materials with high bioactivity are composites, it might be concluded that the beneficial effects on cell survival, proliferation and differentiation are mediated largely by ionic dissolution products such as silica [97,111] or phosphate ions [112,113]. According to this, it has been postulated that best results of osteogenic differentiation of osteoblast progenitors along with bone formation may be expected when calcium phosphate-based materials dissociate easily to calcium and phosphate ions [113]. 

### 3.3. Chromium

The physiological function of chromium in human is currently under debate. Though some cellular functions of chromium have been reported, in 2014, the European food safety authority officially removed it from their list of essential micronutrients [114,115]. The impact of chromium exposition on osteoblasts was investigated in several studies, whereby only toxic effects, causing reduced DNA, RNA and protein synthesis, were reported [116,117]. Furthermore, chromium suppressed collagenase activity in osteoblasts, which reduced collagen formation and deposition and also negatively affected new bone formation [117].

In the field of reconstructive medicine, cobalt-chromium (CoCr) is one of the main alloys used for total hip arthroplasty. However, the Co^2+^ ion released from CoCr surfaces has been reported to severely impact mesenchymal stem cells by altering osteogenic gene expression, affecting osteogenic lineage differentiation and compromising the mineralization process [118]. The impairment of bone formation by chromium and cobalt was further analyzed by the effect of these ions on the expression of various TGF-β isoforms and mineralization in MG-63 and Saos-2 osteosarcoma cells, as well as in primary human osteoblasts. While Co^2+^ decreased the expression of different TGF-β isoforms in all investigated cell types, Cr^3+^ had no impact. Cr^3+^, on the other hand, strongly inhibited the mineralization process of these cells in vitro, whereas Co^2+^, within the range of the tested concentrations, showed no inhibitory effects on mineralization [119]. 

### 3.4. Cobalt (Co^2+^)

As cobalt is a compound of cobalamin, it is an essential trace element, which stimulates the production of red blood cells and promotes angiogenesis by activating hypoxia-inducible transcription factors (HIF) [120,121,122]. Previous studies have demonstrated a rather unfavorable effect of Co^2+^ ions released from CoCr surfaces, affecting osteogenic lineage differentiation of hMSCs, TGF-β isoform expression in osteoblasts and the mineralization process. Conversely, recent data indicate that the impaired mineralization reported by Schröck and colleagues was caused by Cr^3+^ ion release, rather than by Co^2+^ ions [118,119]. 

The angiogenic capacities of cobalt ions sparked the idea of incorporating this metal into different materials used for bone healing in order to stimulate vascularization of implanted grafting materials and thereby enhance the remodeling processes and supporting overall regeneration. The impact of Co^2+^ ions incorporated into calcium phosphate (CaP) coatings for poly-lactic acid (PLA) particles on new blood vessel formation was studied in an intramuscular implantation model in goats. The inflammatory reaction, following a 12-week implantation course, demonstrated no pathologic differences between PLA particles coated with solely CaPs or those coated with Co^2+^ containing CaPs. Formation of blood vessels was significantly increased when Co^2+^ containing CaP-coated PLA particles was implanted, and vessel size was notably increased, suggesting a positive impact of Co^2+^ on vascularization in vivo [123]. 

The impact of Co^2+^ containing CaPs on osteoporotic alveolar bone regeneration was further investigated in rats. Biocompatibility assessment of the material was approved for epithelial Caco-2 and osteoblastic MC3T3-E1 cells, whereby no toxic effects were observed in Caco-2 cells; however, a considerable decrease in cell viability and impairment of cytoskeletal organization was observed in the MC3T3-E1 cells. Despite the negative impact of Co^2+^ ions on osteogenic cells, hydroxyapatite (HAp) nanoparticles doped with Co^2+^ demonstrated dose-dependent acceleration of osteogenesis, osteoporotic bone regeneration and graft material substitution in comparison to HA-nanoparticles without Co^2+^. The authors listed several hypotheses for their observations, including increased transport of Ca^2+^ ions into the extracellular fluids facilitated by the moderate toxicity of Co^2+^ ions as well as increased cytokine production and release, which could potentially boost aminopeptidase activity together with migration and proliferation of endothelial cells [124]. 

The combination of Co^2+^ HAp nanoparticles with blood or plasma rich in growth factors (PRGF) was shown to induce the generation of large quantities of osteoblasts, increase mineralization and accelerate bone regeneration [124]. Taking into consideration that recent studies demonstrated impaired growth factor expression and osteogenic lineage determination in hMSCs exposed to Co^2+^, these observations seem reasonable, as blood and PRGF may compensate for this lower expression, thus enabling proper osteogenic lineage differentiation [119]. Furthermore, the study indicates that bone minerals containing scaffolds, as presented in this study, are suitable for cobalt incorporation, as cobalt does not impair but rather seems to support the mineralization process [119,124]. 

Similar findings were reported by another group, who developed a hydrogel with incorporated Co^2+^ ions. Hydrogels solely doped with Co^2+^ did not increase the amount of regenerated bone volume, bone surface and bone surface density in a rat model in vivo, whereas the addition of BMP2 to the hydrogel did. The observed gain was even more pronounced with the simultaneous loading of Co^2+^ and BMP2 onto the hydrogel, which again favors the hypothesis of a synergistic effect of Co^2+^ in conjunction with growth factors in graft vascularization and bone regeneration [125]. Increased collagen deposition, new bone formation and bone hardness were also reported for cobalt-containing bioglasses compared to bioglasses without cobalt in critical size defects in a rabbit’s femur in vivo [126]. Additionally, the authors showed that the inclusion of both strontium and cobalt into the bioactive glasses even further ameliorated the bone regeneration process.

### 3.5. Copper

While Cu^2+^ is in the most stable oxidation state in aqueous solution, it can also be present as Cu^+^ in the human body exhibiting diverse properties and functions [127]. Together with iron and zinc, copper is one of the most important metals for humans and is especially needed to generate Cu-proteins that have enzymatic functions. Cu-proteins have three main functions in living organisms, such as participation in electron-transfer reactions, transport of oxygen and transport or storage of the metal itself. 

Therefore, copper is involved in multiple physiological functions, including the regulation of bone metabolism and turnover. Cu imbalances also affect the nervous system and can lead to vascular abnormalities in the human body. The impact of copper deficiency on skeletal growth and development was previously assessed in several studies [128,129]. Copper became a material of interest in the field of bone regeneration due to its antibacterial properties and its ability to stimulate collagen fiber deposition and angiogenesis, which represents the first step towards the formation of vital and vascularized tissue [130,131,132]. The effect of copper-doped silicate bioceramics on vascularization was subjected to several studies. Kong and colleagues [133] recently reported a positive impact on the expression of angiogenic growth factors in human umbilical vein endothelial cells (HUVECs) and human dermal fibroblasts (HDFs) in response to Cu^2+^ released from copper silicate bioceramics. Thus, the release of Cu^2+^ ions from porous matrices like bioactive glass should facilitate the ingrowth of bone into the scaffold matrix [134].

Current data support enhanced osteogenic differentiation of mesenchymal stem cells mediated by copper supplementation. Early studies on the effect of copper on MSCs derived from postmenopausal women demonstrated reduced proliferation, a 2-fold enhancement of differentiation into osteoblasts and increased calcium deposition, while alkaline phosphatase activity was considerably diminished in these cells and shifted to an earlier timepoint [135]. Similar findings on the suppression of alkaline phosphatase activity mediated by copper exposition were observed in rat MSCs by Li and colleagues, who reported a clear reduction in osteogenic differentiation of rat MSCs concomitant with the reduction of several osteogenic genes, alkaline phosphatase activity and bone nodule formation. In addition, cytoskeletal abnormalities during osteogenesis were found in these cells. The process of ectopic bone formation in a rat model was also significantly impaired by the presence of copper and while vascularization in the regenerated soft tissue was promoted, collagen formation was strongly inhibited [136]. 

These findings are supported by a study conducted with pre-osteoblastic MC3T3-E1 cells cultured on copper containing bioglasses. While no effects on proliferation and alkaline phosphatase activity of these cells were noted with scaffolds doped with 0.4 wt.% to 0.8 wt.% CuO, 2.0 wt.% showed a significant reduction effect on both. In an in vivo approach, rat calvarial defects showed that a higher concentration of Cu^2+^ ions also substantially reduced new bone formation from 46 ± 8% to 0.8 ± 0.7%, while lower concentrations showed no such impairment. On the other hand, the authors found a stimulatory effect on blood vessel formation dependent of the copper content of the scaffolds, with the greatest impact seen for the highest concentration of 2.0% CuO [137]. Benefits of copper supplementation in the regeneration of critical-sized calvarial defects in rats were further reported by the comparison of chitosan scaffolds and chitosan scaffolds doped with copper. Analysis of micro-CT scans after 4 weeks of healing indicated twice the amount of bone volume in the defects treated with copper containing chitosan scaffolds as compared to scaffolds without copper [138]. 

### 3.6. Gallium (Ga^3+^)

Gallium is a metal that serves no known essential functions in human. While currently being investigated in cancer treatment because of its anti-proliferative properties resulting from the interference with iron-dependent cellular functions, studies also demonstrated that short-term gallium treatment reduces bone turnover in vivo and increases the calcium content of bone in patients suffering from cancer-related hypercalcemia [139]. Furthermore, gallium has the potential to disrupt microbial iron utilization by interacting with iron-binding bacterial molecules called siderophores. In this manner, gallium downregulates bacterial iron uptake and impairs bacterial growth [140]. Gallium-EDTA-coated titanium chips exhibited significant antimicrobial activity against *Escherichia coli* for more than 28 days after coating, underscoring a promising application of gallium-based coatings for effective prevention of biofilm formation, and these chips could be used in dental and orthopedic reconstructive surgery [141]. Additionally, gallium-coated titanium implants showed superior antibacterial properties in vivo and consequently, more effective prevention of biofilm formation than silver coatings [142].

Several studies analyzed the effect of gallium administration on osteoclasts and osteoblasts. While osteoclastic lineage differentiation and resorption activity were lowered by gallium, no impact on viability and proliferation of osteoblasts was noted [143]. In an in vivo approach using a rabbit femoral defect model, gallium-loaded calcium phosphate cements showed no superiority over calcium phosphate cements without gallium in terms of bone healing, whereby the authors implied that no effect was observed due to the low resorption of the material and consequently, low release of Ga^3+^-ions [144]. In a subsequently conducted study, the gallium release from Ga-CaP was optimized and re-evaluated for its beneficial properties in bone healing. Upregulation of osteoblastic marker expression was observed in primary human osteoblasts cultured on the Ga-CaP, whereby late osteoclastic markers were downregulated in primary human monocytes that were previously induced towards the osteoclast lineage. 

The in vivo properties of Ga-loaded CaPs in new bone formation were assessed in a murine bone defect healing model; aside from an enhanced total defect-fill, Ga-CaPs also promoted the synthesis of mature organized collagen [145]. With respect to the current literature, gallium holds a set of promising qualities for future applications in tissue engineering.

### 3.7. Iron (Fe^2+^)

Iron is one of the most important ions in the human organism as it is essential for a variety of cellular processes [146,147,148]. Different cellular effects such as the synthesis of deoxyribonucleic acid (DNA) and ribonucleic acid (RNS), proteins, electron transport processes, cellular proliferation and differentiation are related to iron ions [149,150]. These effects are based on the involvement of iron ions mainly as components of enzyme molecules, such as oxidases, catalases, peroxidases, aconitases, ribonuleotide reductases and nitric oxide synthases, amongst others [150,151,152]. As a coordinating ion in the center of hemoglobin and myoglobin, iron is an essential trace element, required for oxygen transport and regulation of several metabolic enzymes [153,154]. Iron is the loosely bound ion component of the procollagen proline hydroxylase and the procollagen lysine hydroxylase [155]. Both enzymes effect the hydroxylation of proline and lysine residues in the precursors of collagen. Large amounts of iron released from iron-containing implants, however, may cause excessive iron levels in the blood. Here, the free iron can react with peroxides and trigger the formation of free radicals, which are highly reactive and damage lipids, proteins, DNA as well as cellular structures [156,157]. Additionally, hemochromatosis has been demonstrated to result in osteoporosis mediated by increased ferroxidase activity of ferritin. In vitro experiments demonstrated inhibition of osteogenic lineage differentiation in human osteoblasts concomitant with decreased calcification caused by iron overload [158,159,160]. In vivo experiments in zebrafish larvae demonstrated that the mechanism by which iron-overload causes impaired osteoblast function and mineralization is based on the increased generation of reactive oxygen species. Application of deferoxamine, an iron chelator capable of removing whole-body iron, ameliorated the iron-induced negative effects on osteoblastic marker expression and mineralization [161]. Similarly, this was also observed for hepcidin, a regulator of iron-uptake, which is also capable of removing whole body iron. Likewise, hepcidin downregulation elevates the iron level and causes iron-overload mediated interference with osteogenesis [162].

Iron exposure to human bone marrow mesenchymal stem cells (BMSCs) decreased their differentiation towards the osteogenic lineage as well as extracellular matrix mineralization with a total block of lineage commitment at a concentration of 50 µM. In vivo experiments in mice were able to reproduce these findings. The inhibitory effect of iron, however, was specific for osteogenic lineage differentiation, whereas no impact on chondrogenesis and adipogenesis was noted [163]. Furthermore, the promotion of osteoclast formation mediated by iron was previously reported, which additionally underscores the unfavorable features of iron for the purpose of biomedical tissue engineering [164]. In contrast to these previous results, Wang and colleagues reported a positive impact of iron oxide nanoparticles (IONPs) on the osteogenic differentiation of human BMSCs in vitro mediated by MAPK signaling. The authors speculated that the negative impact of iron on osteogenesis observed in previous studies resulted from increased reactive oxygen species (ROS) formation and ferritin activity, whereby this process is proposed to be prevented by nanoparticle formulations [165]. Moreover, Zhao and colleagues analyzed both effects of excessive and low body iron conditions on osteoblast activity [166]. The results showed that an increased iron concentration inhibited osteoblastic activity in a concentration-dependent manner, while a mild iron deficiency led to an increase in cellular activity. In contrast, a severely low iron level completely inhibited osteoblastic differentiation. An enhanced osteoclast formation is one result of an increased iron concentration while osteogenic stimuli are blocked under the same conditions [167]. Thus, further studies will have to clearly determine the potential benefits of iron in tissue engineering.

### 3.8. Lithium (Li^+^)

Lithium is a non-essential trace element and consequently fulfills no known functions in the human organism. However, due to its beneficial impact in the treatment of psychological disorders, lithium has been widely introduced into medical applications [168]. Among the various mechanisms of action that have been proposed for lithium, the stimulation of neural progenitor cell proliferation by the Wnt/β-catenin pathway, which leads to an increase of the brains grey matter, is widely accepted [169,170]. Interestingly, the proliferation of other cell types such as MSCs is also regulated by the Wnt/β-catenin pathway, suggesting that lithium might also modulate the proliferation of these cells [171]. In fact, a recent study reported increased proliferation of hMSCs stimulated by lithium-mediated Wnt/β-catenin signaling in vitro [172]. Additionally, previous studies reported this pathway to be a main regulator of osteoblastogenesis, which made lithium application in the field of tissue engineering even more appealing [173]. Though few studies reported a beneficial impact of lithium supplementation on bone mineral density and a reduction of the risk of fracturing, the molecular mechanisms by which lithium facilitates these effects are not yet completely understood [174,175]. In a transcriptome-based approach used to identify the impact of lithium on osteoblastogenesis, Satija and colleagues reported diminishing proliferation of hMSCs treated with lithium and decreased expression of adipogenic and osteoclastogenic factors accompanied by the induction of osteoblastogenic markers associated with collagen-1 deposition and mineralization; similar results were also reported by other groups [176,177,178]. Systemic lithium application exhibited beneficial effects on bone healing following distraction osteotomy in the tibia of rats. Bone mineral density, the quantity of newly formed mature bone tissue and bone mass regeneration were increased in rats who received a lithium solution through gastric gavage in comparison to those receiving a saline solution, pointing to accelerated callus ossification and bone healing mediated by lithium [179].

To further utilize the beneficial effects of lithium on bone regeneration, various biodegradable lithium-containing scaffolds have been developed and tested for their potential in bone regeneration; preliminary experiments on lithium release, toxicity and osteoblastic cell activity on such scaffolds were promising [180,181]. In vitro experiments comparing pure HAp with lithium-doped HAp scaffolds demonstrated increased osteoblast activity, resulting in accelerated material degradation, and the degradation products exhibited no toxic impacts on osteoblasts and enhanced osteoblast proliferation. Additionally, compressive strength testing revealed favorable mechanical properties of lithium-doped HAp scaffolds [182]. Further evidence on the beneficial impact of lithium incorporation into calcium phosphate cement scaffolds on bone healing was recently demonstrated. Lithium release from this material stimulated the proliferation and differentiation of osteoblasts in vitro by Wnt/β-catenin activation. Application of lithium-doped calcium phosphate cements significantly increased osteogenesis and defect repair in vivo and showed superior osteoconduction and osteointegration compared to pure calcium phosphate cements [183]. Overall, the literature emphasizes that lithium regulates growth and development of osteogenic progenies while suppressing osteoclast development; identification of the exact mechanisms of lithium orchestrating either differentiation or proliferation of osteoblasts represents a pivotal goal for future clinical applications. Nonetheless, lithium seems to directly regulate and benefit osteogenic lineage cells, whereas other metallic ions, such as copper and cobalt, seem to impact bone regeneration by their impact on endothelial cells and accelerated vascularization.

### 3.9. Magnesium (Mg^2+^)

Magnesium is an alkaline earth metal that belongs to group 2 metals of the periodic table. The mammalian body consists of approximately 0.4 g magnesium/kg body weight [184]. More than 90% is bound and stored in bone, muscle and non-muscular soft tissue [184,185], while only a small amount (1–5%) [185] resides in extracellular fluids [186] in the form of ionized/free magnesium (55–70%) or is bound to proteins and anions [184]. 

Magnesium is an important intracellular cation [185,186,187] as it is cofactor for more than 300 enzymatic reactions, essential for the synthesis of proteins and nucleic acids [185,188] and for the transport of both, potassium and calcium ions [185]. Magnesium is also crucial for transphosphorylation of ATP, and changes of intracellular magnesium levels might influence several pathways [189]. 

As magnesium maintains bone strength [185] and bone formation capacity, [184] adequate dietary magnesium plays a major role in musculoskeletal health and is relevant to prevent osteoporosis [190]. A magnesium deficiency exerts negative effects on rat bone metabolism, systemic bone mass [191], and contributes to osteoporosis in humans [189]. It has been proposed that the effects of magnesium deficiency might be the result of increased levels of TNFα, IL-1 [192], and NF-κB ligand (RANKL), along with decreased serum levels of osteoprotegerin (OPG) [193]. 

According to the superior role of magnesium in cellular functions, magnesium-based materials are regarded as promising candidates for bone replacement therapies due to the stimulation capacity of bone cell differentiation in vitro [194,195,196,197] and bone formation in vivo [198,199,200,201]. Currently available materials include different magnesium-containing compounds such as oxides, phosphates and silicates that are used as bone cements, bone scaffolds or implant coatings. Overviews of the different magnesium-based materials—such as bioceramics, e.g., magnesium phosphates (MgO-P_2_O_5_), calcium magnesium phosphates (CaO-MgO-P_2_O_5_), and magnesium glasses (SiO_2_-MgO) [202] are given in recent systematic reviews [203,204,205,206,207,208,209,210,211,212,213,214,215].

Numerous in vitro studies focus on the effects of magnesium ions on bone cells, in terms of enhancing proliferation and migration as well as alkaline phosphatase (ALP) activity of human osteosarcoma MG-63 cells [216], increasing viability and differentiation capacity of a human osteoblast cell line (hFOB1.19, ATCC) [217], cell proliferation of bone marrow derived stromal cells (BMSC), and expression of α2 und α3 integrins [218]. However, additional data provide evidence that the effects of magnesium ions develop dose-dependently [217]. Concentrations of about 1–3 mM Mg^2+^ stimulate gap junctional intercellular communication (GJIC) of osteoblasts [217], while viability, proliferation and differentiation of human BMSCs are ensured by concentrations in the range of 2.5–10 mM [216,218,219,220].

In contrast, decreased mineralization capacity and matrix deposition of BMSCs have been observed in response to magnesium concentrations higher than 1.3 mM Mg^2+^ [221,222,223]. According to the role of magnesium as a physiological calcium antagonist [222], it has been suggested that magnesium substitution for calcium in hydroxyapatite structure [224] and/or modulations of intracellular calcium oscillations with consecutive suppression of spontaneous ATP release and inactivation purinergic receptors are responsible for the decreased mineralization capacity of the cells [221]. Additionally, magnesium has a competitive role against Matrix gla protein (MGP), suggested as a potent inhibitor of HAp crystal growth during mineralization [225]. These results are consistent with emerging studies demonstrating significant suppression of mitochondrial accumulation of calcium ions in MSCs [222] and inhibition of excess calcium-induced mineralization in response to high extracellular magnesium [226]. Similarly, decreased intracellular calcium concentration and decreased calcium influx have been observed when MSCs have been cultured in the presence of a high magnesium concentration [223]. Competition between calcium and magnesium ions for the same ion transporters, such as transient receptor potential cation channel, subfamily M, member 7 (TRPM7) [223] and/or inhibition of expression of calcium-sensing receptor (CaSR) [226], might be responsible for the decreased mineralization capacity. In terms of how high concentrations of Mg^2+^ ions modulate bone cell metabolism and bone cell function, the Wnt/β-catenin anti-calcifying pathway and the magnesium transporter SLC41A1 have been shown to be involved in magnesium-mediated signaling of BMSCs [223]. 

The benefit of biodegradability is that it avoids a second surgery for implant removal and prevents formation of foreign body giant cells in close vicinity of permanent implants, and this has been designated as a major advantage of the magnesium-based materials [227]. The architecture and pore structural conditions of magnesium-enriched scaffolds greatly influence bone formation and remodeling activities [228]. Hydrogen gas released during degradation of magnesium-enriched scaffolds enlarges pre-existing pores, and expands the space for invading cells and blood vessels [201]. Given these beneficial effects, magnesium-based materials have emerged as a new class of biodegradable biomaterials for bone tissue engineering—referred to as next-generation biomaterials [227].

However, considering the rapid degradation rates, magnesium-based implants are still not commonly used in clinical practice [212,227,229]. The “high magnesium microenvironment” created by rapid corrosion of magnesium alloys might disturb calcium-dependent processes and physiology of the cells localized in close vicinity to the implants [222]. Therefore, the balance between calcium and magnesium ions is not only crucial for bone physiology [222] but also for successful osseointegration of magnesium-based materials. 

Additionally, due to rapid corrosion rates, magnesium-based implants have the risks of structural failure and toxic responses immediately after implantation [227]. In the course of degradation, magnesium hydroxide and hydrogen gas are produced, both of which cause detrimental effects on cells and tissue localized close to the implant [188,230]. Controllable in vivo corrosion rates, in terms of establishing sufficient corrosion protection methods at different levels might represent promising tools to overcome these disadvantages [188,212,227,229,230].

### 3.10. Manganese (Mn^2+^)

Manganese is an essential element and is crucial for the proper function of a multitude of enzymes in living organisms [231]. Divalent cations such as Mn^2+^ are also known to influence cell migration by modulating focal adhesion organization via integrins and actin stress fiber formation [232,233]. These properties make manganese an interesting candidate for improving ingrowth and integration of bone grafts and other implantable materials alike. The impact of manganese on MG-63 osteoblastic cells was evaluated in order to confirm this theoretical benefit of manganese supplementation in the process of new bone formation. Manganese supplementation reduced cell proliferation, migration, ERK/MAPK-signaling and collagen I as well as alkaline phosphatase expression in a dose-dependent manner. Interestingly, the mRNA level of bone sialo protein (BSP) was increased by manganese exposure, whereas the BSP protein level was not elevated [234].

Interestingly, doping alumina tubes with manganese significantly enhanced tissue maturation and osteogenesis in vivo in rats; the authors noted that the surface structure of the alumina tubes was altered by manganese incorporation which made it impossible to distinguish whether the observations resulted from the phase composition or the surface topography modification [235]. However, manganese is also reported to have insulin-mimetic properties and other properties within this class, such as VAC-increased fracture site vascularization by local application, which led to the hypothesis that manganese might also accelerate fracture healing [236,237]. In fact, a group reported a significant increase in the mechanical properties of bone, mineralized tissue formation and VEGF-expression in a rat femoral fracture model when manganese chloride (MnCl_2_) was supplemented. Additionally, blood vessel density was dramatically increased by MnCl_2_ treatment, suggesting increased vascularization, fracture healing and osteogenesis, implicating a potential function for manganese in tissue engineering [238].

### 3.11. Silver (Ag^+^)

Due to its antimicrobial properties, silver has a long history of application for medical purposes, whereas the investigation of potential functions of silver in bone regeneration is a quite recent occurrence [239]. Analysis of the tissue response to silver acetate-coated Dacron vascular grafts implanted into the dorsal skinfold chamber in mice revealed higher functional capillary density without affecting inflammatory host tissue response, collagen formation, apoptosis and cell proliferation as compared to uncoated grafts [240]. Furthermore, functionalization of silver nanoparticles in tissue regeneration has already been introduced into commercially available wound dressings, as they exhibit outstanding anti-microbial and anti-inflammatory properties [241,242,243]. Additional arguments for the utilization of silver nanoparticles instead of other silver formulations like silver nitrate in tissue engineering were recently reported by Quin and colleagues [244]. They showed that the lowest toxic concentration of silver nanoparticles in urine-derived stem cells was substantially higher than that assessed for silver nitrate. More interestingly, however, was the reported promotion of osteogenic lineage induction and actin polymerization of these cells, which was only observed for AgNPs, and not for AgNO_3_ [244]. In fact, the stimulatory impact of AgNPs on the mineralization of MC3T3-E1 osteoblastic cells maintained by miRNA-mediated increased expression of genes associated with bone formation was previously reported [245].

In order to identify putative impacts of AgNPs in the process of osteogenic lineage induction, the entire transcriptome of MC3T3-E1 cells in response to AgNP exposure was analyzed. The authors found that, aside from the upregulation of different bone morphogenic proteins important for osteogenesis, the enhancement of osteoclastic marker expression was the most pronounced transcription-based alteration [246]. Based on the stimulatory properties of AgNPs on keratinocyte proliferation and migration and fibroblast differentiation, which contributes to the promotion of wound contraction, the impact of AgNPs on proliferation and differentiation of MSCs was analyzed [247,248]. AgNPs successfully promoted MSC proliferation and osteogenic differentiation in vitro. In vivo experiments using a femoral fracture model in mice support the preliminary observations, as AgNPs encapsulated in collagen were able to accelerate callus formation and fracture gap closure. Though the exact impact of AgNPs in this process remains elusive, the authors suggested that the possible chemotactic impact of AgNPs on MSCs and fibroblasts, as well as induction of MSC proliferation and osteogenic differentiation, was responsible for the observed effects [249]. Despite the here reported beneficial impacts of AgNPs on hard-and soft-tissue related cells, further studies will have to elucidate the clinical practicability relevance of AgNPs application in the promotion of osteogenesis.

### 3.12. Strontium (Sr^2+^)

Strontium (Sr) is an alkaline earth metal and belongs to the group 2 elements of the periodic table. Although it is considered as a non-essential element, there is growing interest concerning the effects of Sr on cells of the bone. This interest is based upon the fact that strontium ranelate has been used in Europe as a therapeutic drug for the treatment of osteoporosis since 2004. Osteoporosis is a serious systemic skeletal disorder and is becoming a major health problem due to rapid population aging. As osteoporosis leads to dramatic changes of the skeleton in terms of markedly decreased bone mass and reduced bone quality, as well as altered architecture at the macroscopic and microscopic levels, the disease is associated with a high incidence of osteoporotic fractures. 

The use of Sr for the treatment of osteoporosis is based upon its dual mode of action: Sr influences both osteoblasts and osteoclasts and gives rise to increased bone formation capacity of osteoblasts and decreased bone resorption activity of osteoclasts [250,251,252,253,254]. Due to its similarity to calcium, the effects of Sr are largely mediated by the calcium sensing receptor (CaSR), which is a membrane-bound receptor expressed in osteoblasts and osteoclasts [255,256,257,258]. In response to Sr, intracellular signaling pathways are activated, resulting in enhanced proliferation and differentiation of mesenchymal stem cells and osteoblasts along with increased mineralization and deposition of extracellular matrix [250,255,259] by activating the Wnt/Catenin signal pathway [250,260]. Additionally, in response to the activation of this pathway, OPG (osteoprotegerin) levels of osteoblasts and their precursors increase, whereas RANKL (receptor activator of nuclear factor κB ligand) expression of the cells decreases [261]. The expression patterns in favor of OPG suppress differentiation of osteoclasts and limit the extent of bone resorption. Similar effects are observable in the course of direct interaction of Sr with the extracellular domain of the CaSR: downstream cascades stimulate diacylglycerol (DAG9-protein kinase C (PKC) βII which in turn induces osteoclast apoptosis [257]. In a recent in vitro study, Sr could be detected by means of mass spectrometry within the cytoplasm of osteoclasts which were cultivated in combination with a Sr-enriched calcium phosphate cement. Cell differentiation of the osteoclasts was therefore delayed [262]. However, the mechanism by which the ions enter the cells, and to what extent intracellular Sr deposition influences cell signaling, must still be clarified. 

Besides the beneficial effects on bone metabolism, systemic administration of strontium ranelate increases the risk of cardiovascular diseases [263]. Therefore, its use is restricted to patients who show no signs of heart and circulatory diseases.

For the benefit of osteoporotic patients and in light of the effects of Sr on bone remodeling, combinations of Sr with bone substitutes might represent a successful approach to overcome the adverse effects of systemic administration of strontium ranelate. Accordingly, Sr is used for apatite coatings of orthopedic and dental implants [264,265,266], and is incorporated into different bone cements [262,267,268,269,270,271,272,273]. Because of their subsequent substitution by natural bone in the course of physiological remodeling, it has been proposed that calcium phosphate-based cements ensure the local release of Sr [274], and therefore might represent ideal bone substitutes for the osteoporotic bone. According to this suggestion, stable incorporation of Sr into the crystal lattice of the bone mineral is based upon remodeling activities of osteoblasts and osteoclasts (for a review see [275]), and Sr uptake is especially high in newly formed bone tissue [276]. So placed at the disposal of the bone cells, Sr might locally regulate their activities as well as the bone healing process in the course of further remodeling.

### 3.13. Vanadium (V^+^)

Vanadium is a trace element present in basically all living organisms and is predominantly stored within the bone tissue [277]. Because of its growth factor mimicking properties, it was previously suggested that vanadium might positively influence osteogenesis [278,279]. An early study analyzing the impact of vanadium derivatives on osteoblast-like UMR106 cells reported enhanced proliferation, alkaline phosphatase activity and even differentiation [280]. As insulin supplementation ameliorates negative effects of diabetes on bone regeneration and local insulin treatment enhances fracture healing in healthy rats, the insulin-mimetic properties of vanadium are currently being investigated as a safe and cost-efficient alternative to insulin supplementation [281,282]. 

Intramedullary delivery of an organic vanadium salt (vanadyl acetylacetonate) in a rat femoral fracture model significantly promoted cell proliferation, vascular endothelial growth, callus cartilage formation and mineralization and considerably increased torque to failure compared to treatment with saline control solutions [236]. A vanadium-loaded collagen scaffold was recently described by Cortizo and colleagues; although vanadium loading increased membrane permeability, no changes in the collagen structure were observed. Furthermore, attachment, growth and osteoblastic as well as chondrocytic differentiation of rBMPCs was improved by loading vanadyl acetylacetonate onto collagen membranes [283]. Vanadium coating of titanium implants was also shown to enhance fibroblast attachment and proliferation, which suggests potential benefits in soft tissue healing by vanadium treatment [284]. Taken together, published data demonstrate vanadium to be an interesting metal with great potential in regulating both angiogenesis and osteogenesis; however, further studies are required to support these preliminary findings.

### 3.14. Zinc (Zn^2+^)

Zinc is an essential trace element that is pivotal for proper immune system functioning, cell division and for skeletal development and therefore has been implemented into biomaterials for orthopedic and dental applications [285,286,287]. Furthermore, zinc and zinc alloys are promising biomaterials as load-bearing scaffolds as they have similar mechanical properties to mammalian bone, especially Zn^2+^ ions, which have a multitude of physiological functions. Zinc led to increased ECM mineralization in hMSC culture by promoting the expression of ALP and osteopontin [288]. Also, with respect to SMCs, a concentration-dependent behavior was found in the presence of Zn^2+^ in vitro. In the range 80–120 μM, a change in biological response was observed by inhibition of viability and proliferation [289]. When Zn was used in different titan coatings, the measured expression of Zn-transporters (ZnT1 and ZIP1) suggested that cells prefer Zn^2+^ present at the biomaterial interface rather than plain diffusion of Zn^2+^ ions in the surrounding medium [290]. Additional studies on the actions of zinc supplementation in osteogenesis reported enhanced collagen deposition and mineralization of osteoblast like MC3T3-E1 cells, antagonizing effects on osteoclastogenesis with simultaneous promotion of osteoblastogenic differentiation and increased osteoblast activity mediated by zinc supplementation in a concentration-dependent manner [291,292,293]. Zinc phosphate-loaded barrier membranes showed excellent anti-microbial properties, capable of inhibiting bacterial colonization upon membrane exposure and avoiding potential infections [294]. To further analyze beneficial properties of zinc in GBR procedures, cross-linked gelatin membranes loaded with zinc hydroxyapatite powder were compared to cross-linked collagen membranes in a rat calvarial defect model. After a period of 6 weeks, bone defect fill was 80 ± 2%, 60 ± 5% and 40 ± 2% for the zinc-loaded gelatin membrane, the collagen membrane and the unfilled control group, respectively, demonstrating the tremendous potential for the application of zinc in bone regeneration approaches [295]. Antibacterial effects, excellent biocompatibility and stimulatory impact on the activity of osteoblast-like MG63 cells were also recently reported for nanocomposites of carboxylated graphene oxide sheets decorated with zinc oxide nanoparticles, emphasizing the potential application of zinc in nanoparticle formulations for tissue engineering [296]. 

Zinc ions released from zinc-doped tricalcium phosphates were able to enhance TRAP and ALP activity of hBMSCs and to regulate multinuclear giant cell formation and activity of RAW264.7 macrophages [297]. De novo bone formation in a canine ectopic implantation model was only induced by the addition of zinc to TCPs, however, not by TCPs alone, whereby the rate of new bone formation was coherent with zinc concentration [297]. Zinc is also an attractive candidate for the development of coatings in order to promote the integration of implants. Regarding this matter, a study analyzed rBMSC activity in response to zinc-loaded titanium oxide coatings and the impact of zinc-supplementation on osseointegration in a rat implantation model. In comparison to TiO_2_ coatings without zinc, osteogenic gene expression was upregulated in rBMSCs cultivated on zinc-doped TiO_2_ coatings, and early-stage new bone formation as well as bone contact ratio were increased in vivo [290]. Yu and colleagues further reported increased osteogenic differentiation and mineralized matrix deposition in rat bone marrow-derived pericytes (BM-PCs) and significant promotion of new bone formation around titanium implants in osteopenic rabbits with the application of zinc-modified calcium silicate coatings. Molecular analysis revealed that zinc exerts these actions by regulating the TGF-β/Smad signaling pathway, which is pivotal for osteoblastogenesis [298]. Reports on zinc in biomedical applications for tissue engineering, especially with regards to the positive impacts on osteoblastogenesis, osteoblast activity and tissue mineralization, are promising for improving implant osseointegration, accelerating bone regeneration and inhibiting biofilm formation.

### 3.15. Others

There are other metals and their corresponding ions which have been demonstrated to have an effect on the bone regeneration process [195]. Webster et al. have shown a higher adsorption of calcium, vitronectin and collagen on yttrium-doped HAp [299]. Others including zirconium and also molybdenum are used in different metal alloys which are used for orthopedic and dental applications [300]. The latter metals are primarily used to achieve specific material properties. There are additional metals that play a role as implant materials, especially titanium, which builds up a very stable oxide layer, and thus, can be considered almost inert under physiological conditions [301]. Nevertheless, for titanium and its alloys, it was shown that released titanium enhanced the release of bone resorbing cytokines from LPS-stimulated monocyte cultures [302]. Long-term in vivo studies in baboons revealed an increased titanium ion concentration in urine as well as enhanced levels in tissues [303]. Nevertheless, no toxic effects were observed up to 8 years after implantation.

## 4. Conclusions

The existing bone substitute materials only provide osteoconductive healing capacities, and most of the newly developed tissue engineering strategies are still not applicable in the daily clinical routine. The presented overview of the physiological mode of action of different metal ions and their influence on the process of bone tissue regeneration has shown that their addition to existing bone substitute materials may alter the inflammation and foreign body response or the onset of bone regeneration as well as material durability. Another important problem is the availability and the cost of suitable bone grafting material for the increasing need of an aging population.

It is obvious that different parameters play an important role in the use or the combination of metals with existing biomaterials. Furthermore, it has been demonstrated that the concentration of the released metal ions plays a crucial role for the bone formation process. Thereby, it would be beneficial to have the ions present in close vicinity of the implanted biomaterial, as bone regeneration should preferably occur directly at the implant site. On the other hand, metals can be incorporated into scaffolds, which support a continuous release to support early induction of osteoblast differentiation, as they can control transcriptional regulators like Runx2 and therefore osteogenesis.

There is still ongoing work investigating specific effects as well as possible synergistic effects of metal ions with other synthetic materials on the differentiation into osteogenic lineage. Therefore, it is necessary to plan and run additional experiments and studies in almost every scientific field to develop the suitable biomaterial patients need.
